# Management of Acute Sore Throat in Community Pharmacies: Insights from a Survey of Italian Pharmacists

**DOI:** 10.3390/pharmacy14050110

**Published:** 2026-07-25

**Authors:** Rachele Aspesi, Paolo Levantino, Giulia Ciancarella, Andrea Nacci, Pietro Tasegian, Diego Maria Michele Fornasari

**Affiliations:** 1 Ordine dei Farmacisti di Varese, 21100 Varese, Italy; 2 Federazione Nazionale Associazioni Giovani Farmacisti (Fenagifar), 00199 Roma, Italy; paololevantino@gmail.com; 3 SIMG (Italian College of General Practitioners and Primary Care), Lazio Regional Section, 00196 Roma, Italy; 4 ENT, Audiology, and Phoniatrics Unit, University of Pisa, 56126 Pisa, Italy; 5 SIMG (Italian College of General Practitioners and Primary Care), Umbria Regional Section, 06012 Città di Castello, Italy; 6 Department of Medical Biotechnology and Translational Medicine, Università degli Studi di Milano, 20122 Milan, Italy

**Keywords:** community pharmacy, pharyngitis, survey

## Abstract

**Background**: Acute sore throat (pharyngitis) accounts for 10–30% of ambulatory visits annually. Bacterial etiology, primarily Group A Streptococcus, is confirmed in only 20–34% of cases, and inappropriate antibiotic administration remains frequent, contributing to antimicrobial resistance. Community pharmacists play a key role in symptomatic management and stewardship, but real-world practices remain underexplored. **Methods**: A 16-item online questionnaire surveyed 629 Italian community pharmacists via professional networks, covering demographics, diagnostic tools, symptom assessment, treatment preferences, and follow-up. Responses were analyzed using descriptive statistics and interpreted within a multidisciplinary expert framework. **Results**: Italian community pharmacists (*n* = 629), predominantly mid-career and experienced individuals, prioritize targeted symptom questioning (flu-like signs (59.96%), fever (48.73%), pain intensity (49.64%)) and medical referral over formal diagnostic tools like the Centor criteria (unused in 53.66% of cases). A total of 46.74% report performing a Strep A test in fewer than 20% of patients. Topical sprays (83.33%) and lozenges (44.69%), especially containing flurbiprofen (94.19%), dominate recommendations, followed by systemic analgesics (45.79%). For mild cases, anti-inflammatory/analgesic sprays/lozenges (35.42%) and antiseptic lozenges (35.61%) dominate, while in severe cases, drugs with anti-inflammatory or analgesic effects are often preferred (72.47%). Most pharmacists request a follow-up (73.22%). **Conclusions**: Italian community pharmacists report a predominantly topical-first approach focused on symptomatic treatment and referral when appropriate. Diagnostic gaps and inconsistent follow-up represent actionable targets. The findings inform training outputs prioritizing simplified triage, expanded symptom checklists, spray/lozenge optimization, and 3–5-day call-backs. This work aims to promote pharmacists’ antimicrobial stewardship to reduce antibiotic use while enhancing patient-centred sore throat care in Italy’s pharmacy network.

## 1. Introduction

Acute sore throat (pharyngitis) is usually caused by viral infections. Beyond pain, common symptoms include dysphagia, fever, and cough, and it is often associated with cervical lymphadenopathy. Sore throat represents one of the most common reasons for primary care consultations worldwide, accounting for 10–30% of ambulatory visits annually, with bacterial etiology—primarily Group A Streptococcus (GAS)—confirmed in only 20–34% of cases depending on seasonal and regional conditions. Beyond bacteria, viral and non-infectious aetiologies are also possible [1,2]. While most episodes are self-limiting, inappropriate antibiotic administration (or self-administration) remains a problem and facilitates the phenomenon of antibiotic resistance among common pathogenic bacteria [1]. Community pharmacists, as first-line healthcare providers, play a pivotal role in symptomatic management, educated counselling, and referral to a physician for specialized care [2], particularly in systems like Italy’s, where pharmacy access is widespread.

Current clinical guidelines recommend targeted diagnostic strategies, including clinical scores (e.g., Centor or McIsaac criteria) and rapid antigen detection tests (RADTs) [3,4], and advise restricting antibiotics to high-risk or confirmed bacterial cases. In uncomplicated presentations, topical symptomatic treatments represent an effective first-line approach, providing rapid local relief while limiting unnecessary systemic drug use and inappropriate antibiotic consumption.

However, real-world data describing how Italian community pharmacists assess and manage sore throat in daily practice are lacking. This evidence gap limits the development of targeted training initiatives and antimicrobial stewardship strategies tailored to the pharmacy setting.

Furthermore, sore throat management in Italy is not standardized and does not follow any specific diagnostic–therapeutic pathway. Furthermore, previous efforts mainly revolved around the role of the hospital pharmacist as key figure of the antimicrobial stewardship, whereas the community pharmacist should have a pivotal role in this process too [5,6]. Indeed, in Italy, antimicrobial stewardship is increasingly promoted through regional and primary-care initiatives, and community pharmacies are considered an early access point for education and information of the patients. Moreover, the literature indicates that AMS is increasingly becoming a structured undergraduate teaching subject [7]. Additionally, antibiotics are prescription-only medicines in Italy, and dispensing them without a prescription is explicitly illegal, even if some noncompliance has been reported in community practice [8].

In this context, pharmacy-based RADTs for Strep A can support more appropriate referral and antibiotic use, but they do not replace the physician’s clinical decision; in Italy these tests are typically paid by the patient or set by local pharmacy/regional arrangements rather than by a universal reimbursement scheme. Furthermore, published evidence shows that RADTs generally have high specificity but variable sensitivity, so a negative result does not fully exclude Strep A and should be interpreted in the clinical context [9].

Therefore, this study aimed to investigate Italian community pharmacists’ approaches to the assessment, treatment, and follow-up of acute sore throat and to identify opportunities to optimize patient care and progress towards the strengthening of antimicrobial stewardship within community pharmacies.

## 2. Materials and Methods

A cross-sectional observational study based on a questionnaire was conducted to describe real-world practices in managing acute sore throat in Italian community pharmacies. The study had descriptive and exploratory objectives and followed CROSS guidelines for reporting the results [10].

Questionnaire development

A multidisciplinary expert panel composed of two general practitioners, one otolaryngologist, one clinical pharmacologist, and two community pharmacists developed a 16-item multiple-choice questionnaire. Items were designed based on current clinical guidelines and relevant studies in the literature and covered four domains: demographics, diagnostic approaches, therapeutic recommendations, and follow-up practices. The questionnaire was reviewed for clarity and content relevance by the panel prior to dissemination. The expert panel did not influence data collection or aggregation.

Participants and recruitment

The inclusion criteria comprised licenced pharmacists registered with the professional order and practicing in community pharmacies in Italy. The online questionnaire was developed by EDRA SpA using the free software SurveyMonkey. The invitation was sent by e-mail to 45,000 Italian pharmacists registered in an online, EDRA-managed, platform, MediKey (https://ssl.medikey.it/about.aspx, accessed on 29 June 2026), between October and December 2025.

Access to the platform was restricted to registered pharmacists, thereby excluding individuals who are not licenced or not actively practicing. No incentives were offered for participation, and consent was implied through the voluntary completion of the survey.

Data collection

The questionnaire was administered online between October and December 2025 using a secure survey platform.

Statistical analysis

Data were provided as excel sheets and analyzed using descriptive statistics. Categorical variables are reported as frequencies and percentages using Microsoft Office 365 Excel software. Given the exploratory nature of the study, no inferential analyses were planned. Statistical analyses were performed using standard spreadsheet software. In total, we obtained responses from 629 participants. In the results, “n” represents the number of responses obtained for each item. Not all the participants completed the survey answering to all the 16 questions. In these cases, valid answers were nonetheless considered in the final analysis.

Ethical considerations

Participation was voluntary and anonymous. According to national regulations for non-interventional survey-based studies, formal ethical approval was not required.

## 3. Results

Respondent Characteristics

The pharmacists’ survey responses (*n* = 629) provide a snapshot of common management practices for patients seeking relief from sore throat symptoms in Italian community pharmacies (a full table of questions and answers is provided in Table 1). The respondents were predominantly mid-career and experienced pharmacists. Indeed, the most represented age ranges were 46–55 years (25.91%, *n* = 163/629), followed by 56–65 years (25.44%, *n* = 160/629), with 13.51% being over 65 (*n* = 85/629). Consequently, our cohort reported significant experience in the role of community pharmacist, with 40.07% of participants reporting spending >25 years in their careers (*n* = 246/614), followed by 21–25 years (15.15%, *n* = 93/614). Targeted patient questioning was the most frequent strategy (98.48%, *n* = 582/591), far exceeding indicated product dispensing (32.49%, *n* = 192/591) or following patients’ requests (8.12%, *n* = 48/591). Medical referral was common (46.87%, *n* = 277/591), indicating the propensity to carefully manage potentially severe cases, even in absence of a final diagnosis. The Strep A test is reportedly recommended by a small percentage of pharmacists (14.04%, *n* = 83/591). Furthermore, by the results of our survey, we can hypothesize that this attitude is not affected by the number of years of experience in the pharmacy setting (Table S1).

Diagnostic Practices

The Centor criteria or similar validated scores revealed low uptake: 53.66% of pharmacists never applied them (*n* = 308/574), 39.20% applied them only selectively (*n* = 225/574), and just 7.14% applied them consistently (*n* = 41/574). Similarly to previous observations, years of experience did not have a significant influence on the answers (Table S1).

Moreover, rapid pharyngeal Strep A tests were infrequently reported, with 46.74% of pharmacists performing them in <20% of cases (*n* = 265/567), 13.23% performing them in 20–50% of cases (*n* = 75/567), and 38.45% citing absent on-site instruments/apparels to perform the test (*n* = 218/567). Higher frequencies (>50%) were rare (1.59%, *n* = 9/567), reflecting logistical barriers over attitudinal resistance.

Symptom Assessment

Among symptoms to explore, flu-like signs were in the lead (59.96%, *n* = 331/552), followed by fever (48.73%, *n* = 269/552), pain intensity (49.64%, *n* = 274/552), and dysphagia (40.04%, *n* = 221/552). Fewer individuals prioritized prior medications (23.19%, *n* = 128/552) or comorbidities (33.88%, *n* = 187/552). Interestingly, a sub-analysis of the responses revealed that those that answered negatively to Q4 (use of the Centor criteria; Table 1) still indicated flu-like symptoms and pain intensity as primary signs to look for when facing a patient with sore throat (Table S1).

Treatment preferences

Topical sprays topped the recommendations (83.33%, *n* = 455/546), alongside oral systemic analgesics/anti-inflammatories like paracetamol/ibuprofen (45.79%, *n* = 250/546) and lozenges (44.69%, *n* = 244/546). Traditional herbal medicinal products were recommended in 12.45% of cases, whereas mouthwashes and homeopathic remedies were recommended less frequently (1.83% and 3.30% of cases, respectively). These recommendations were primarily driven by the presence of mild symptomatology (50.28%, *n* = 273/543) and greater manageability compared to systemic drugs (20.81%, *n* = 113/543). Simple administration (12.34%, *n* = 67/543) or patient preference (10.50%, *n* = 57/543) were also considered, though to a lesser extent. Reports of adjunct use were minor (6.08%, *n* = 33/543), positioning topicals as the predominant application for uncomplicated cases.

The choice of a particular topical product is influenced differently by several factors: active ingredient class (antiseptic vs. anti-inflammatory: 39.59%, *n* = 213/538) and symptom profile (33.64%, *n* = 181/538) guided selections over pharmaceutical form (16.36%, *n* = 88/538) or patients’ preference (10.41%, *n* = 56/538), emphasizing the need to follow a pharmacological rationale in decision-making. Specifically, flurbiprofen emerged as the most frequently recommended active ingredient (94.19%, *n* = 503/534), followed by herbals (35.58%, *n* = 190/534), benzydamine/cetylpyridinium (29.21%, *n* = 156/534), and ketoprofen (16.48%, *n* = 88/534). This consensus highlights its perceived dual anti-inflammatory/analgesic superiority for pharyngitis.

Regarding the pharmaceutical form, sprays were deemed most effective/handy (61.05%, *n* = 326/534), followed by lozenges (34.83%, *n* = 186/534), while mouthwashes did not receive any preference. For severe sore throat, anti-inflammatory sprays/lozenges were indicated as the most frequently used (72.47%, *n* = 379/523), and antiseptics, medical devices, or herbal products were marginally used (2–6%). However, topical products were still recommended if flu-like symptoms (47.70%, *n* = 249/522) or moderate pain (22.03%, *n* = 115/522) were present, and they were used in combination with other treatments in a lower percentage of cases (29.69%, *n* = 155/522).

Regarding combination therapy, the surveyed pharmacists reported that topicals frequently get paired with systemics, prioritizing anti-inflammatory/analgesic compounds (54.97%, *n* = 282/513), followed by lenitives (21.64%, *n* = 111/513) and immunostimulants (16.18%, *n* = 83/513).

Follow-up practices

Last, follow-up was requested by 73% of pharmacists (most after 3 days: 37.38%, *n* = 194/519), with 26.78% abstaining from this practice (*n* = 139/519), suggesting proactive monitoring. However, even though follow-up at 3 days was the most frequently indicated answer (37.38%, *n* = 194), the rest of the positive answers were divided between follow-up at 2 days, 5 days, or “maximum 1 week”, indicating the need to standardize this step.

## 4. Discussion

Acute sore throat constitutes a leading cause of primary care visits globally, with bacterial etiology—predominantly Group A Streptococcus (GAS)—verified in only 20–34% of cases, yet antibiotic overprescription endures, exacerbating resistance [11,12].

In this framework, pharmacists’ “sentinel” position in health systems grant them a pivotal role in providing patients with the necessary advice to face their disorders with improved awareness. Indeed, several international studies have already analyzed pharmacists’ involvement in sore throat management. In Wales, the Sore Throat Test-and-Treat service showed that a pre-specified pathway followed by pharmacists ensured appropriate use of antibiotics and absorbed a substantial workload that would otherwise end up in other healthcare settings [13]. A similar study conducted in Poland concluded that a similar model was also likely to be feasible [14]. Finally, UK audits (NHS PQS) and Canadian GAS guidelines highlight the role of pharmacy-driven management of sore throat [15]. This falls within the framework of the global test-to-treat movement, which strongly relies on the role of pharmacists as primary health care providers, particularly in rural areas [16].

However, similar analyses and/or recommendations for the Italian landscape are not yet available. Hence, this survey of 629 Italian community pharmacists offers a comprehensive snapshot of real-world practices and lays the foundation for further action.

**Figure 1:**
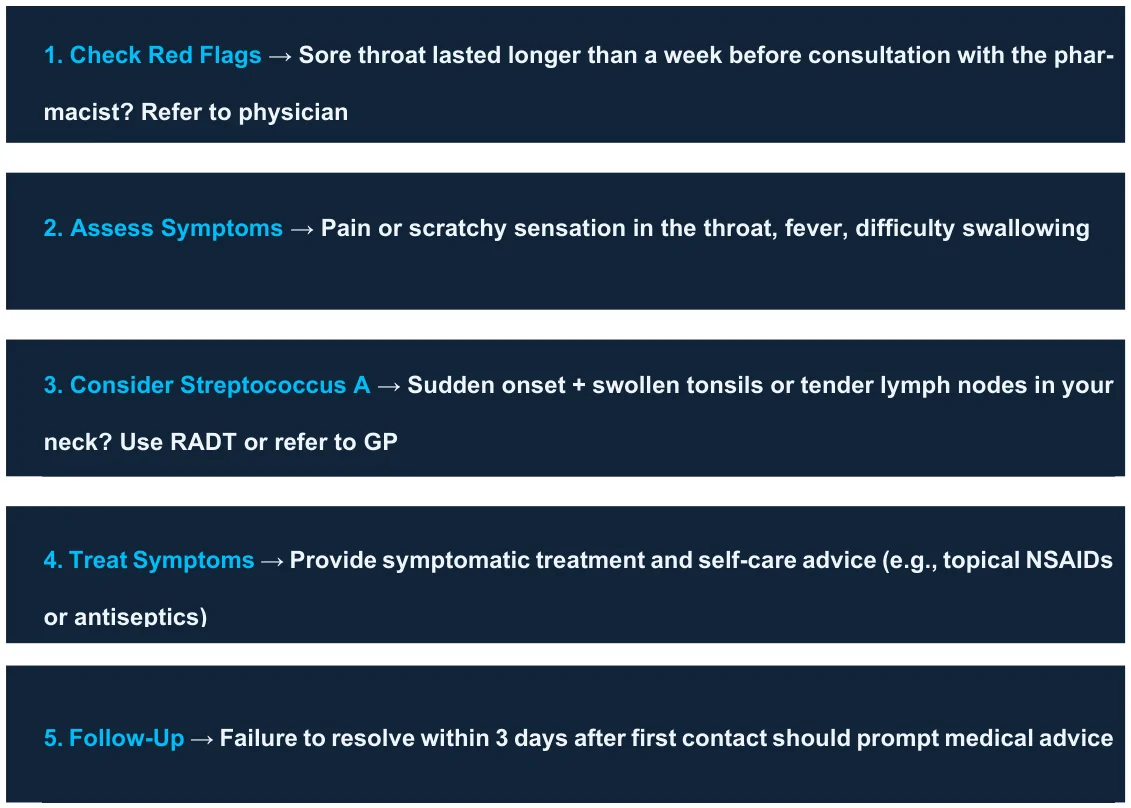
A simple approach for managing sore throats in pharmacies, aligning with previous recommendations by the International Pharmaceutical Federation [22].

Our analysis critically revealed a lack of utilization of validated scales and/or criteria in patients’ counselling. Indeed, over half of the surveyed participants never employed the Centor criteria, probably since these criteria are not routinely incorporated into pharmacists’ undergraduate education in Italy or, possibly, due to inherent limitations of the use of clinical scores alone, which do not possess the specificity and sensitivity of laboratory tests [17]. Furthermore, RADTs were only conducted in <20% of cases; this is partly explained by the absence of necessary on-site equipment, which makes it impossible to perform the test. Moreover, this diagnostic gap may limit pharmacists’ ability to consistently identify patients who need medical referral, and thus should be considered when evaluating the safety of a symptomatic-first approach. These results also suggest that simplified, practical screening tools (Figure 1) could be distributed in pharmacies to facilitate initial sore throat management between self-limiting viral pharyngitis and high-risk scenarios that will likely require medical attention. This approach would enable more complete alignment with current guidelines and recommendations, empowering pharmacists to perform effective initial screening and, as already described in other states, could lead to resource optimization and increased therapeutic appropriateness [13,14].

When analyzing therapeutic decision-making, the current practice reflects a logical stepwise progression, with topical agents such as sprays and lozenges emerging as the preferred forms for symptomatic relief, and flurbiprofen is the preferred active ingredient. Moreover, for mild sore throat, pharmacists frequently recommend anti-inflammatory/analgesic sprays or lozenges and antiseptic lozenges, and for severe cases, anti-inflammatory/analgesic sprays or lozenges represent the primary option in our survey. This is coherent with the published literature, which supports the use of lozenges and sprays due to easy administration and demonstrated tolerability [18,19]. Systemic oral analgesics/anti-inflammatories serve as adjuncts, especially when there is evidence of severe sore throat. This survey also suggests that pharmacists evaluate key indicators when counselling a patient and evaluating sore throat characteristics. It is important to note that the duration of sore throat, though only acknowledged by roughly one third of the participants, should be rigorously taken into account during sore throat evaluation as it may indicate the need to reassess the patient and/or use a different therapeutic approach [16]. Indeed, acute sore throat is usually self-limiting with a mean duration of around seven days, and a lack of improvement after 3–4 days is explicitly cited as an indication for clinical re-evaluation [20,21].

Cohort data from over 14,000 adults show that persistent or non-resolving symptoms are associated with higher reconsultation rates and suppurative complications such as peritonsillar abscess, suggesting that duration is an independent marker of risk beyond baseline pain intensity [21]. Indeed, both international [23] and Italian guidelines (SIMG 2024) similarly flag “worsening of symptoms after 3 days” (even under antibiotics) or prolonged persistence as alarm signs for clinical reassessment, antibiotics, further investigations, or specialist referral. Integrating symptom duration into pharmacists’ decision-making would enable more precise patient stratification, helping to identify those requiring closer follow-up or changes in therapeutic strategy [24]. Notably, even though pharmacists prioritized assessing flu-like symptoms (59.96%) over patient comorbidities (33.88%), this “pragmatic” approach might lead to the oversight of crucial risk factors in vulnerable populations, such as immunocompromised or elderly patients, where comorbidities significantly influence management decisions.

Active ingredients guide selections, with antiseptics or analgesics endorsed for topical use. Among active ingredients, flurbiprofen emerged as the most frequently dispensed in the pharmacies. This molecule has already proven its efficacy and safety as a local NSAID for rapid relief of pain due to sore throat, irrespective of the etiology [25,26,27].

An interesting finding concerns the lack of preference for mouthwashes, which indicates a lower recommendation for their use compared to other pharmaceutical forms. The use of topical herbal/phytotherapeutic medicines drops sharply from mild to severe cases, indicating severity-appropriate escalation, yet this trend invites scrutiny of evidence for “natural” alternatives amid commercial influences and patient demand.

Our survey also highlights a significant finding regarding follow-up practices. While most pharmacists report encouraging behaviours, some aspects are worth further consideration. Indeed, nearly three-quarters of respondents recommend a follow-up, primarily to reassess symptom persistence and guide potential referral. When considering the optimal time frame, the majority converge between 3 and 5 days, in line with evidence-based recommendations for sore throat reassessment. However, a smaller proportion opt for earlier or more generic time points (“within one week”). Notably, one in four pharmacists does not request any follow-up, potentially increasing the risk of self-medication or delayed identification of complicated cases. Even considering the limitations of a survey study, these findings may underscore the need to standardize patient review protocols, promoting a 3–5-day follow-up window to strengthen the pharmacist’s monitoring role and reinforce antimicrobial stewardship. This is particularly relevant in certain contexts, like sore throat management, where adherence to recommended treatment prevents complications such as peritonsillar abscess and emergence of drug-resistant bacteria. Previous studies have reported that pharmacist-led interventions in chronic diseases (such as COPD/asthma and type 2 diabetes mellitus) improve therapy compliance [28,29,30].

A further education and formation opportunity may stem from the analysis of combination therapies. In these cases, there is a net preference for systemic anti-inflammatories alongside topicals, supplemented by lenitives or immunostimulants—often prophylactically patient-requested—underscoring opportunities for education on appropriate adjuncts.

Notably, we did not report any substantial differences in management practices according to years of experience, supporting the hypothesis that our findings, specifically low usage of the Centor criteria and RADT, were not influenced by the higher number of mid-career and senior pharmacists in our cohort. This finding suggests that the observed approaches to acute sore throat management are relatively consistent across professional seniority levels. However, this was not the primary scope of our work and these hypotheses were not tested with statistical tests.

Structural factors may instead play a more relevant role in shaping practice patterns. In particular, in our survey, the limited availability of rapid antigen detection tests within Italian pharmacies emerged as a key constraint, potentially influencing diagnostic strategies independently of individual experience. Future studies specifically designed to explore geographic, urban–rural, and organizational factors may help to further elucidate the determinants of practice variation in community pharmacy settings.

Some limitations of this study need to be acknowledged. First, we did not conduct a formal questionnaire validation process, pilot testing, or reliability analyses. Moreover, this survey was conducted from October to December 2025, which typically coincides with the peak season for respiratory infections in Italy. This timing could introduce recall bias or seasonal bias, potentially influencing their reported practices, symptom assessment priorities, and perception of case severity. Second, we need to take into account the response rate that we obtained (629/45,000; 1.4%) cannot represent the opinion of the entire professional class of Italian community pharmacists.

We also reported a discrepancy between the low use of standardized diagnostic tools and the relatively high rate of medical referrals, but the study design did not allow us to determine whether referrals were guided by diagnostic testing, informal clinical criteria, or the pharmacist’s professional judgement. Additionally, the descriptive nature of this work provides a snapshot of the Italian pharmacy practice but does not include a deeper statistical analysis of the findings. We also acknowledge that the sample is skewed toward mid-career and senior pharmacists, with limited representation of younger professionals, which may affect the generalizability of our observations. Finally, further studies could involve other healthcare professionals or investigate patients’ knowledge and adherence to treatment recommendations.

## 5. Conclusions

This survey shows that pharmacists predominantly adopt a topical-first management strategy, favouring sprays and lozenges with anti-inflammatory or analgesic properties (particularly flurbiprofen), and use systemic medications or medical referral for persistent cases. The Centor criteria and RADTs are still underused. Hence, the distribution of rapid tests for sore throat evaluation in every pharmacy could increase the use of such tools. Additionally, educational initiatives could refine symptom evaluation (including duration), optimize topical treatment choices, and standardize 3–5-day reassessment.

## Figures and Tables

**Table 1:** Questions, response options and results of the questionnaire. Data in the parentheses represent raw number of responses and percentages of total, respectively.

Question (Q)	Response Options
Q1: What is your age group? (629 respondents)	- 25–35 years (69; 10.97%) - 36–45 years (152; 24.17%) - 46–55 years (163; 25.91%) - 56–65 years (160; 25.44%) - ≥65 years (85; 13.51%)
Q2: How many years have you worked in a pharmacy? (614 respondents)	- Less than 5 years (45; 7.33%) - 5–10 years (84; 13.68%) - 11–15 years (81; 13.19%) - 16–20 years (65; 10.59%) - 21–25 years (93; 15.15%) - More than 25 years (246; 40.07%)
Q3: Which of the following approaches do you most frequently adopt with sore throat patients? (select top 2 most relevant) (591 respondents)	- Ask the patient more targeted questions to explore symptoms and recommend the most appropriate treatment (582; 98.48%) - Dispense a product indicated for sore throat based on the condition (192; 32.49%) - Dispense a product requested by the patient for symptom management (48; 8.12%) - Suggest a rapid pharyngeal test for Streptococcus A (83; 14.04%) - Refer to medical attention when appropriate (277; 46.87%)
Q4: In assessing sore throat symptoms, do you use validated clinical tools, such as Centor criteria? (574 respondents)	- Yes, always (41; 7.14%) - No, never (308; 53.66%) - Only in some cases (225; 39.20%)
Q5: In what percentage of patients do you perform the rapid pharyngeal test for Streptococcus A? (567 respondents)	- <20% (265; 46.74%) - 20–50% (75; 13.23%) - 50–70% (8; 1.41%) - >70% (1; 0.18%) - I do not have the means to perform this test in the pharmacy (218; 38.45%)
Q6: Which of the following aspects do you consider most appropriate to explore with the patient to understand sore throat characteristics? (select top 3 most relevant) (552 respondents)	- Pain intensity (274; 49.64%) - Difficulty swallowing (221; 40.04%) - Sore throat duration (179; 32.43%) - Presence of fever (269; 48.73%) - Presence of cough, cold, or other flu-like symptoms (331; 59.96%) - If they have already taken other medications (128; 23.19%) - Smoking history or other relevant conditions (e.g., allergies, gastroesophageal reflux, chronic diseases) (187; 33.88%) - None (0; 0%)
Q7: Which product types do you most frequently recommend for symptomatic sore throat treatment? (select top 2 most relevant) (546 respondents)	- Oral sprays (455; 83.33%) - Tablets (244; 44.69%) - Mouthwashes (10; 1.83%) - Oral systemic analgesics/anti-inflammatories (e.g., paracetamol, ibuprofen, ketoprofen) (250; 45.79%) - Medical devices (40; 7.33%) - Herbal or phytotherapeutic products (68; 12.45%) - Dietary supplements (7; 1.28%) - Homeopathic remedies (18; 3.30%)
Q8: Which of these factors would most encourage you to recommend a topical symptomatic drug for sore throat? (543 respondents)	- Mild symptomatology (273; 50.28%) - Greater ease vs. systemic oral drug (113; 20.81%) - Ease of administration (67; 12.34%) - Patient preference (57; 10.50%) - Only as adjunct to ongoing systemic symptomatic therapy (33; 6.08%)
Q9: When recommending a topical symptomatic drug for sore throat, which characteristic most influences your product choice? (538 respondents)	- Active ingredient class (e.g., antiseptics vs. anti-inflammatories) (213; 39.59%) - Pharmaceutical form (e.g., spray vs. tablets vs. mouthwashes) (88; 16.36%) - Symptom characteristics (181; 33.64%) - Patient request/preference (56; 10.41%)
Q10: Which topical active ingredients or drugs for sore throat do you most frequently recommend? (select top 2 most relevant) (534 respondents)	- Flurbiprofen (503; 94.19%) - Plant-based medicines (190; 35.58%) - Benzydamine hydrochloride, Cetylpyridinium chloride (156; 29.21%) - Ketoprofen (88; 16.48%) - Benzydamine hydrochloride (73; 13.67%) - 2,4-Dichlorobenzyl alcohol, Sodium benzoate (29; 5.43%) - 2,4-Dichlorobenzyl alcohol, Amylmetacresol (29; 5.43%)
Q11: For topical products, which pharmaceutical form do you consider most effective and/or manageable for sore throat treatment? (534 respondents)	- Tablet (186; 34.83%) - Spray (326; 61.05%) - Mouthwash (0; 0%) - None in particular (22; 4.12%)
Q12: For mild sore throat, which topical product would you most frequently recommend? (528 respondents)	- Spray/tablet with anti-inflammatory/analgesic (187; 35.42%) - Antiseptic tablet (188; 35.61%) - Anti-inflammatory/analgesic mouthwash (4; 0.76%) - Antiseptic mouthwash (6; 1.14%) - Medical devices (30; 5.68%) - Herbal or phytotherapeutic products (111; 21.02%) - Phytotherapeutic dietary supplements (2; 0.38%) - Does not recommend other topical products (0; 0%)
Q13: For severe sore throat, which topical product would you most frequently recommend? (523 respondents)	- Spray/tablet with anti-inflammatory/analgesic (379; 72.47%) - Antiseptic tablet (12; 2.29%) - Anti-inflammatory/analgesic mouthwash (13; 2.49%) - Antiseptic mouthwash (3; 0.57%) - Medical devices (32; 6.12%) - Herbal phytotherapeutic products (6; 1.15%) - Does not recommend other topical products (78; 14.91%)
Q14: Do you tend to recommend topical products for sore throat in combination with other treatments? (522 respondents)	- Always/almost always (155; 29.69%) - Only in cases of moderate-severe pain (115; 22.03%) - Also in presence of flu-like and cold symptoms (249; 47.70%) - Never (3; 0.57%)
Q15: If you recommend a topical drug to the patient, do you ask them to return to the pharmacy for follow-up? If yes, after how long? (519 respondents)	- I never ask for patient follow-up (139; 26.78%) - Yes, after 2 days (64; 12.33%) - Yes, after 3 days (194; 37.38%) - Yes, after 5 days (76; 14.64%) - Yes, within a maximum of one week (46; 8.86%)
Q16: If recommending combination therapy with topical treatment for sore throat management, which would likely be your first choice? (513 respondents)	- Oral systemic anti-inflammatory/analgesics (282; 54.97%) - Multivitamin supplements (7; 1.36%) - Phytotherapeutic products (30; 5.85%) - Immunostimulant products (e.g., lactoferrin, echinacea) (83; 16.18%) - Soothing products (e.g., propolis, honey-lemon based products) (111; 21.64%)

## Data Availability

All the data that were generated through the survey have been included in the text or in the Supplementary Data.

## Supplementary Materials

The following supporting information can be downloaded at https://www.mdpi.com/article/10.3390/pharmacy14050110/s1.

## Abbreviations

The following abbreviations are used in this manuscript:

GAS	Group A Streptococcus
RADT	Rapid Antigen Detection Test
