# Supplementary material for: Management of Acute Sore Throat in Community Pharmacies: Insights from a Survey of Italian Pharmacists
Source: Pharmacy (Basel). 2026 Jul 25;14(5):110. doi: 10.3390/pharmacy14050110 (PMC13516034; doi:10.3390/pharmacy14050110)
Supplement: Supplementary file 1 [file pharmacy-14-00110-s001.zip › pharmacy-4349536-supplementary.pdf]

Supplementary Table S1

Age 25-45 (Q1)

Approach (Q3)	n.	%
Ask the patient targeted questions to explore symptoms and recommend the most appropriate treatment	207	98.10
Dispense a product indicated for sore throat based on the condition	81	38.39
Dispense the product requested by the patient for symptom management	24	11.37
Suggest a rapid pharyngeal test for Streptococcus A	24	11.37
Refer to medical attention when appropriate	86	40.76
**Total respondents**	**211**	**100.00**

Age 46->65

Approach (Q3)	n.	%
Ask the patient targeted questions to explore symptoms and recommend the most appropriate treatment	375	98.68
Dispense a product indicated for sore throat based on the condition	111	29.21
Dispense the product requested by the patient for symptom management	24	6.32
Suggest a rapid pharyngeal test for Streptococcus A	59	15.53
Refer to medical attention when appropriate	191	50.26
**Total respondents**	**380**	**100.00**

Responses to Q3

Experience <5–15 years

**Approach**	**n.**	**%**
Ask the patient targeted questions to explore symptoms and recommend the most appropriate treatment	198	97.54
Dispense a product indicated for sore throat based on the condition	83	40.89
Dispense the product requested by the patient for symptom management	22	10.84
Suggest a rapid pharyngeal test for Streptococcus A	23	11.33
Refer to medical attention when appropriate	80	39.41
**Total respondents**	**203**	**100.00**

**Approach**	**n.**	**%**
Ask the patient targeted questions to explore symptoms and recommend the most appropriate treatment	384	98.97
Dispense a product indicated for sore throat based on the condition	109	28.09
Dispense the product requested by the patient for symptom management	26	6.70
Suggest a rapid pharyngeal test for Streptococcus A	60	15.46
Refer to medical attention when appropriate	197	50.77
**Total respondents**	**388**	**100.00**

Responses to Q4, stratified by age or experience

Age 25–45

Response	n.	%
Only in some cases	67	32.37
No, never	128	61.84
Yes, always	12	5.80
**Total respondents**	**207**	**100.00**

Age 46–>65

Response	n.	%
Only in some cases	158	43.05
No, never	180	49.05
Yes, always	29	7.90
**Total respondents**	**367**	**100.00**

Experience <5–15 years

Response	n.	%
Only in some cases	63	32.14
No, never	121	61.73
Yes, always	12	6.12
**Total respondents**	**196**	**100.00**

Experience 16–>25 years

Response	n.	%
Only in some cases	162	42.86
No, never	187	49.47
Yes, always	29	7.67
**Total respondents**	**378**	**100.00**

Responses to Q5, stratified by age (Q1)

Age 25–45

Percentage	n.	%
I do not have the means to perform this test in the pharmacy	83	40.69
20-50%	36	17.65
<20%	82	40.20
>70%	1	0.49
50-70%	2	0.98
**Total**	**204**	**100.00**

Percentage	n.	%
I do not have the means to perform this test in the pharmacy	135	37.19
20-50%	39	10.74

Percentage	n.	%
<20%	183	50.41
>70%	0	0.00
50-70%	6	1.65
**Total**	**363**	**100.00**

Responses to Q6, stratified by Q4

Only in some cases

Aspect to explore	n.	%
Pain intensity	96	44.24
Difficulty swallowing	80	36.87
Sore throat duration	67	30.88
Presence of fever	115	53.00
Presence of cough, cold or other flu-like symptoms	132	60.83
If they have already taken other medications	48	22.12
Smoking history or other relevant conditions (e.g. allergies, gastroesophageal reflux, chronic diseases)	85	39.17
**Total respondents**	**217**	**100.00**

No, never

Aspect to explore	n.	%
Pain intensity	158	53.38

Aspect to explore	n.	%
Difficulty swallowing	129	43.58
Sore throat duration	97	32.77
Presence of fever	133	44.93
Presence of cough, cold or other flu-like symptoms	180	60.81
If they have already taken other medications	69	23.31
Smoking history or other relevant conditions (e.g. allergies, gastroesophageal reflux, chronic diseases)	89	30.07
**Total respondents**	**296**	**100.00**

Yes, always

Aspect to explore	n.	%
Pain intensity	20	51.28
Difficulty swallowing	12	30.77
Sore throat duration	15	38.46
Presence of fever	21	53.85
Presence of cough, cold or other flu-like symptoms	19	48.72
If they have already taken other medications	11	28.21
Smoking history or other relevant conditions (e.g. allergies, gastroesophageal reflux, chronic diseases)	13	33.33
**Total respondents**	**39**	**100.00**
